# Photodynamic therapy and imaging based on tumor-targeted nanoprobe, polymer-conjugated zinc protoporphyrin

**DOI:** 10.4155/fso.15.2

**Published:** 2015-11-01

**Authors:** Jun Fang, Long Liao, Hongzhuan Yin, Hideaki Nakamura, Vladimir Subr, Karel Ulbrich, Hiroshi Maeda

**Affiliations:** 1 Research Institute for Drug Delivery Science, Sojo University, Ikeda 4-22-1, Kumamoto 860-0082, Japan; 2 Laboratory of Microbiology & Oncology, Faculty of Pharmaceutical Science, Sojo University, Kumamoto, Japan; 3 Department of General Surgery, Sheng Jing Hospital, China Medical University, Shenyang City, Liaoning Province, 110004, P. R. China; 4 Institute of Macromolecular Chemistry, Academy of Sciences of the Czech Republic, Prague, Czech Republic

**Keywords:** fluorescent nanoprobe, photodynamic therapy, theranostic nanomedicine, tumor imaging, zinc protoporphyrin

## Abstract

**Aim:**

To evaluate the potential of tumor-targeted nanoprobe, *N*-(2-hydroxypropyl)methacrylamide copolymer-conjugated zinc protoporphyrin (PZP) for photodynamic therapy (PDT) and tumor imaging.

**Materials & Methods:**

Different tumor models including carcinogen-induced cancer were used, PZP was intravenously injected followed by irradiation with xenon or blue fluorescent light on tumor.

**Results:**

One PZP 20 mg/kg (ZnPP equivalent) dose with two or three treatments of light at an intensity of ≥20 J/cm^2^ caused necrosis and disappearance of most tumors (>70%) in different tumor models. We also confirmed PZP-based tumor imaging in carcinogen-induced breast tumor and colon cancer models.

**Conclusion:**

These findings support the potential application of PZP as a tumor-selective nanoprobe for PDT as well as tumor imaging, by virtue of the enhanced permeability and retention effect.

## Abstract (lay abstract)

To evaluate the potential of a tumor-targeted nanoprobe, PZP and normal xenon light source for photodynamic therapy and tumor imaging, different tumor models including cancer induced by carcinogen were used. In all models, a high accumulation of PZP in tumor was found after intravenous injection, resulting in remarkable therapeutic effect. These findings support further research to assess the potential application of PZP as a future nanomedicine for photodynamic cancer therapy and imaging in cancers of the esophagus, breast, lung, colon, rectum, urinary bladder and cervix.

**Figure 1.**
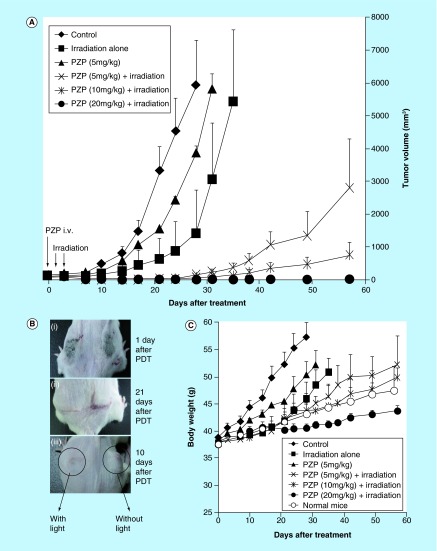
**Photodynamic therapy of S180 solid tumor by using polymeric zinc protoporphyrin and a xenon light source (MAX-303; Asahi Spectra): polymeric zinc protoporphyrin dose dependence.** Different amounts of polymeric zinc protoporphyrin (Znpp) were injected intravenously when tumor diameters measured 5–10 mm. After 24 and 48 h, light irradiation (36 J/cm^2^) was performed. Tumor growth and body weight were determined every 2 or 3 days. **(A)** Suppression of tumor growth by PDT and PZP. **(B)** Macroscopic images of tumors after photodynamic therapy. With 20 mg/kg PZP (ZnPP equivalent), tumors turned pinkish to reddish and then blackish necrotic tissue appeared at 24 h after PDT **(Bi)**; no sign of tumor growth was observed on day 21 after treatment **(Bii)**; in one mouse bearing two tumors, the tumor receiving light irradiation clearly demonstrated shrinkage, whereas the tumor that had not had light treatment manifested no apparent effect **(Biii)**. **(C)** Body weight changes after PDT. Data are means ±SD; n = 5–10. See text for details. PDT: Photodynamic therapy; PZP: *N*-(2-hydroxypropyl)methacrylamide copolymer-conjugated zinc protoporphyrin.

**Figure 2.**
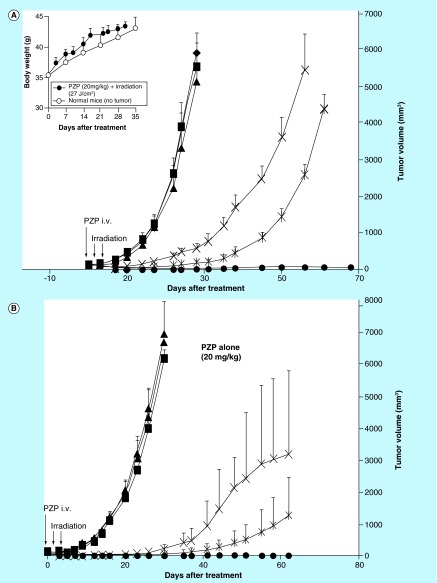
**Photodynamic therapy of S180 solid tumors with polymeric zinc protoporphyrin and a xenon light source: light dose dependence.** **(A)** Mice received treatments with light of different intensities for 5 min. Inset shows the body weight change of mice receiving highest dose of photodynamic therapy (27 J/cm^2^) compared with normal mice without treatment. **(B)** With the total amount of irradiation set at 27 J/cm^2^, we tested different combinations of light intensity and irradiation time. Data are means ±SD; n = 5–10 (see text for details). PZP: *N*-(2-hydroxypropyl)methacrylamide (pHPMA) copolymer conjugated zinc protoporphyrin.

**Figure 3.**
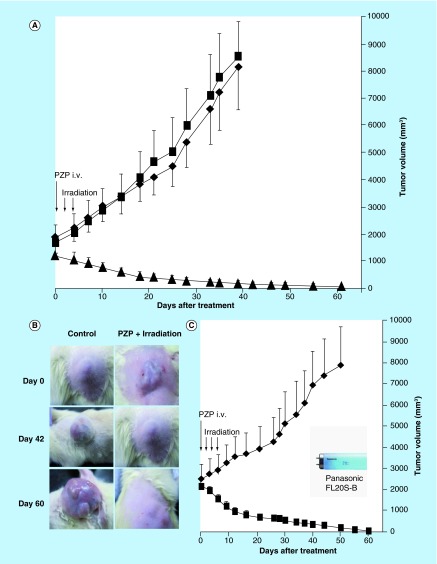
**Photodynamic therapy with polymeric zinc protoporphyrin of 7,12-dimethylbenz[*a*]anthracene-induced breast cancer in rats.** When tumor diameters were 10–20 mm, PZP (20 mg/kg) was injected intravenously, followed after 24 and 48 h by light irradiation. **(A)** A xenon light (MAX-303; Asahi Spectra) was used at 24 and 48 h after PZP administration, with the irradiation dosage being 27 J/cm^2^ (90 mW/cm^2^ for 5 min each time). **(B)** Macroscopic pictures of breast tumors treated as in **(A)**; compared with untreated control rats, in which tumors grew quickly and achieved a diameter of 25–30 mm from a palpable tumor (˜10 mm in diameter) within 40 days, rats receiving this photodynamic therapy (PDT) demonstrated a significant reduction in tumor size; on day 60 after PDT, the tumor disappeared completely, whereas the control tumor (no PDT) continued to grow. **(C)** a blue fluorescent light (Panasonic FL20S-B) with an emission wavelength that matched the absorption band of ZnPP for excitation was used to irradiate the tumors. Rats received an intravenous injection of 20 mg/kg PZP and then 20 J/cm^2^ of irradiation (2.8 mW/cm^2^ for 60 min) at 24, 48 and 72 h after administration of PZP. Data are means ±SD; n = 5–6. See text for details. PZP: *N*-(2-hydroxypropyl)methacrylamide copolymer-conjugated zinc protoporphyrin.

**Figure 4.**
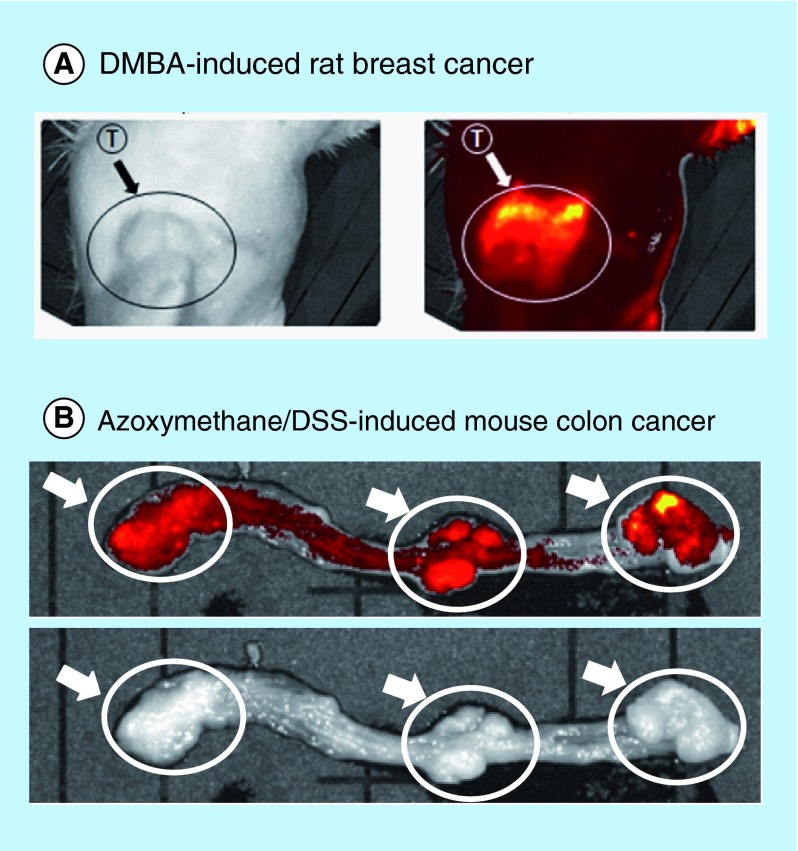
***In vivo* fluorescence imaging of carcinogen-induced tumors with *N*-(2-hydroxypropyl)methacrylamide copolymer-conjugated zinc protoporphyri.** Fluorescent views of DMBA-induced rat breast cancers **(A)** and azoxymethane/dextran sulfate sodium-induced colon cancers **(B)** were obtained at 48 h after intravenous drug injection (15 mg/kg zinc protoporphyrin). Arrows point to fluorescent tumor nodules (see text for details). DMBA: 7,12-dimethylbenz[a]anthracene; DSS: Dextran sulfate sodium; ZnPP: Zinc protoporphyrin; PZP: *N*-(2-hydroxypropyl)methacrylamide copolymer-conjugated zinc protoporphyrin.

**Figure 5.**
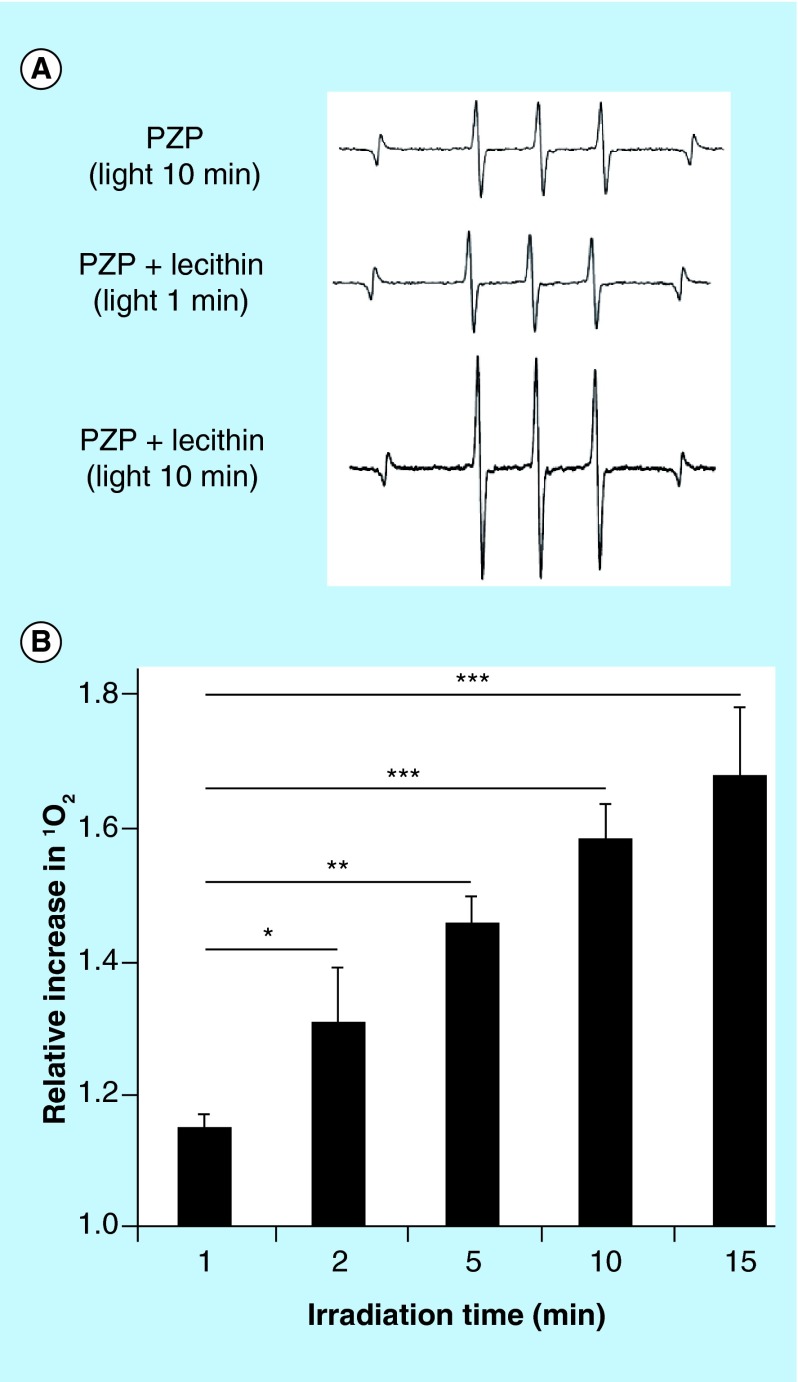
**Generation of singlet oxygen from *N*-(2-hydroxypropyl)methacrylamide-conjugated zinc protoporphyrin exposed to light.** **(A)** Electron spin resonance spectra for PZP in the presence of lecithin after light irradiation for the indicated times. **(B)** Relative increase in the generation of ^1^O_2_ from PZP. Data are means ±SD; n = 4–5 (see text for details). *p < 0.05, **p < 0.01, ***p < 0.001. PZP: *N*-(2-hydroxypropyl)methacrylamide-conjugated zinc protoporphyrin.

Photodynamic therapy (PDT) consists of three components: light, a photosensitizer and oxygen. When photosensitizers are irradiated by a light having an absorbing wavelength, they generate singlet oxygen (^1^O_2_) and other reactive oxygen species (ROS) that kill tumor cells [1–4]. That is, after administration of a photosensitizer, light with the appropriate wavelength is directed at a tumor, where photosensitizers in the tumor become excited and undergo intersystem crossing with molecular oxygen to generate ^1^O_2_. As a group of highly reactive molecules, ROS including ^1^O_2_ have rapid reactions and can damage different biomolecules, including proteins, DNA and lipids, which leads to tumor cell death [5].

To achieve the ideal PDT, one critical issue that remains to be addressed is the selectivity of photosensitizers for tumor tissues. That is, photosensitizers must accumulate selectively in tumor tissues, not in normal tissues. This accumulation would mean generation of toxic ROS exclusively in tumor tissues, and thus minimal side effects in normal tissues and organs. However, conventional or existing photosensitizers, such as porfimer sodium (Photofrin), talaporfin sodium (Laserphyrin) and chlorin, are small molecular weight compounds of less than 1000 Da. Also, many protoporphyrin derivative photosensitizers are quite hydrophobic (difficult to dissolve in water) and tend to aggregate in aqueous systems. Those photosensitizers therefore usually have very poor pharmacokinetic properties, such as a short plasma half-life, and no satisfied tumor-targeted drug accumulation occurs. Despite excellent *in vitro* results with these photosensitizers, *in vivo* therapeutic effects were always less significant. In addition to experiencing the accompanying undesirable side effects, patients who receive these treatments must avoid light to prevent photoactivation reactions in the skin and eyes, because of the indiscriminate distribution of photosensitizers.

During the past few decades, a tumor-targeting strategy was developed with biocompatible macromolecules that utilizes the unique anatomical and pathophysiological characteristics of the blood vasculature of solid tumors. Solid tumors are generally hypervascular and have a defective vascular architecture [6]. Also, vascular mediators that lead to high vascular permeability are extensively produced. Macromolecules larger than 40–50 kDa selectively accumulate and remain in tumor tissues because of their poor lymphatic recovery from tumor tissues, but no such accumulation is seen in normal tissues. This phenomenon is now well known as the enhanced permeability and retention (EPR) effect, which our group first reported in 1986 [7]. The EPR effect has become a gold standard for the development of macromolecular anticancer drugs, so-called nanomedicines, including micelles, polymer conjugates and liposomes [6,8–9]. In the past decades, we and other groups have developed many polymer-conjugated photosensitizers including nanoparticles, micelles, liposomes, that showed improved pharmacokinetics, preferential accumulation in tumor tissues, and thus enhanced *in vivo* PDT effects in animal tumor models [10–16]. And more recently some more advanced delivery systems for PDT such as porphysomes [17] and polymeric photosensitizers with protease-sensitive linkers [18] have been developed. All these findings suggested the potential and significance of EPR-based tumor-targeted PDT.

Zinc protoporphyrin (ZnPP) is a potent inhibitor of heme oxygenase-1 which is known as a key antioxidative/antiapoptotic enzyme and survival factor in tumor cells and also called heat shock protein 32 [19]. We first developed water-soluble polymeric ZnPP micelles, in other wrods, pegylated ZnPP and styrene maleic acid copolymer micelles encapsulating ZnPP, as a potential anticancer agent by targeting heme oxygenase-1 to decrease the antioxidant defense activity of tumor cells and thus induce cell death (apoptosis) caused by oxystress produced by infiltrated macrophages and leukocytes [18–20]. These polymeric forms of ZnPP accumulated selectively in tumor tissues by virtue of the EPR effect, and achieved significant antitumor effects [20–23].

Because many porphyrin derivatives have been used as photosensitizers in PDT, we further expected the potential application of ZnPP in PDT, especially using these polymeric ZnPP for tumor-targeted PDT. We then found significant generation of ^1^O_2_ from polymeric ZnPP and marked PDT effect in various murine tumor models [14,15], suggesting these polymeric ZnPP forms would be useful in PDT. In addition, as a porphyrin derivative ZnPP also has fluorescing capacity, meaning that such tumor selectivity of these polymeric ZnPP could be applied to fluorescent tumor imaging.

Along this line, more recently we synthesized another polymeric ZnPP by using *N*-(2-hydroxypropyl)methacrylamide (pHPMA) copolymer [16]. The resultant pHPMA-ZnPP conjugate (PZP) formed micelles in physiological solutions that had a size of approximately 80 nm and showed superior *in vivo* pharmacokinetics with high tumor-targeting ability [16]. With PZP, we observed a significant PDT effect in a murine sarcoma S180 model. More important, the high tumor-selective accumulation made highly sensitive *in vivo* tumor imaging possible [16].

Here, we report the potential clinical application of PZP as a new photosensitizer for PDT and tumor imaging. We used different tumor models including 7,12-dimethylbenz[*a*]anthracene (DMBA)-induced breast tumor in rats and colon cancer induced by azoxymethane/dextran sulfate sodium (DSS) in mice, both of which are good models of naturally occurring tumors. To investigate the possibility of commonly used light source for PDT, in this study we evaluated normal xenon light and blue fluorescent light as irradiation sources, which fit the maximal absorbance and excitation in the Soret band of ZnPP (i.e., ˜420 nm). We also discuss the possible mechanisms of ^1^O_2_ generation from PZP in tumor cells.

## Materials & methods

### Materials

Protoporphyrin IX, zinc acetate, DSS and egg lecithin were purchased from Wako Pure Chemical, Osaka, Japan. Azoxymethane and DMBA were from Sigma, MO, USA. 2,2,6,6-Tetramethyl-4-piperidone was purchased from Tokyo Chemical Industry, Tokyo, Japan. Other chemicals of reagent grade were from Wako Pure Chemical and were used without further purification.

### PZP

Semitelechelic pHPMA polymer with terminal amino group (mean molecular size ˜12 kDa) was synthesized by RAFT polymerization in the Institute of Macromolecular Chemistry, Prague, Czech Republic and PZP was synthesized, purified and characterized in Sojo University as described previously [16]. The conjugate has a 20% ZnPP loading and shows decreased hydrophobicity/good water solubility, more than 30 mg/ml in water, so that in aqueous solution large micellar particles form with a hydrodynamic diameter of 82.8 ±41.8 nm and a neutral ς potential of +1.12 mV [16].

### Animals, cells & tumor models

Female Sprague–Dawley rats, 5 weeks old, were purchased from SLC, Shizuoka, Japan. Male ddY mice and female ICR mice, all 6 weeks old, were from Kyudo Co., Ltd, Saga, Japan. All animals were maintained at 22 ±1°C and 55 ±5% relative humidity with a 12-h light/dark cycle. All experiments were approved by the animal ethics committees and carried out according to the Laboratory Protocol for Animal Handling of Sojo University.

Mouse sarcoma S180 cells (2 × 10^6^), maintained by weekly passage in mouse ascites, were implanted subcutaneously in the dorsal skin of ddY mice to obtain the S180 solid tumor model.

### Carcinogen-induced tumors

To establish the DMBA-induced breast tumor model, 10 mg of DMBA in 1 ml of corn oil was administered to Sprague–Dawley rats with a sonde via the oral route. In this model, tumors usually appear in the breast after 12–16 weeks. For the azoxymethane/DSS-induced colon cancer model, azoxymethane, 10 mg/kg dissolved in saline, was first administered intraperitoneally to ICR mice; 1 week later, 2% DSS was given orally in the drinking water for 1 week. In this model, tumors usually appear in the colorectal region after 6–8 weeks. In both models described here, tumors grew in nearly all subject animals.

## Evaluation of the PDT effect of PZP in different tumor models

For the S180 tumor model, when tumors achieved a diameter of about 5–10 mm, different concentrations of PZP (5, 10 and 20 mg/kg of free ZnPP equivalent) dissolved in saline were injected intravenously. Tumors were then exposed to 400–700-nm xenon light (MAX-303; Asahi Spectra, Tokyo, Japan) at 24 and 48 h after the intravenous PZP injection because it was known from our previous paper that PZP accumulated significantly in tumor compared with normal tissue at 24 h after intravenous injection and it retained in tumor till at least 48–72 h [16]; various light intensities (30–120 mW/cm^2^) and irradiation times (5–15 min) were utilized, according to the different therapeutic protocols as described in detail in the Results section.

Similarly, for the DMBA-induced breast cancer model, after tumor diameters measured 10–20 mm, which is relatively large size and the tumors could be cleared perceived, PZP, at the concentration of 20 mg/kg (ZnPP equivalent), was injected intravenously via the tail vein. Light irradiation was used as described for the S180 model above. In addition, a blue fluorescent lamp (Panasonic FL20S-B; Panasonic, Osaka, Japan) with wavelength of 420 ±30 nm was used for irradiation of some groups of rats, at 24, 48 and 72 h after PZP injection, for 60 min (2.8 mW/cm^2^) at each time point.

Tumor growth was assessed every 2 or 3 days by measuring the tumor sizes with a caliper. The tumor volume (V) was estimated by measuring longitudinal cross-section (L) and transverse section (W) according to the formula V = (W^2^ × L)/2.

## *In vivo* fluorescence imaging with PZP in cancinogen-induced tumors

When tumors in the above-described rat breast cancer model measured 1–2 cm in diameter, rats were injected intravenously with 15 mg/kg PZP (ZnPP equivalent). At 24 h after injection, rats under anesthesia with isoflurane gas were subjected to *in vivo* fluorescence imaging with the IVIS XR system (Caliper Life Sciences, Hopkinton, MA, USA) (excitation at 430 ±15 nm and emission at 695–770 nm). The light intensity was 7.1 μW/cm^2^ and exposure time was 2 s.

Also, for the murine colorectal cancer model, 10 weeks after azoxymethane/DSS treatment as described above, PZP was injected intravenously at 15 mg/kg (ZnPP equivalent); 24 h after injection, mice were killed and colons were collected. The whole colons were then subjected to *in vivo* imaging with IVIS XR system as just described.

## Electron spin resonance (ESR) spectroscopy

Generation of ^1^O_2_ from PZP exposed to light was analyzed by evaluating ESR spectra, via an ESR spectrometer (JES FA-100; JEOL, Tokyo, Japan) at 25°C. Sample solutions contained 200 μg/ml PZP (40 μg/ml ZnPP equivalent), 20 mM 2,2,6,6-tetramethyl-4-piperidone (spin trapping agent) and various concentrations of lecithin. Samples in flat quartz cells (Labotec, Tokyo, Japan) were irradiated (28 mW/cm^2^) by using xenon light at 400–700 nm (MAX-303; Asahi Spectra) for the indicated times. The ESR spectrometer was usually set at a microwave power of 1.0 mW, amplitude of 100 kHz and field modulation width of 0.1 mT.

## Statistical analyses

Data were analyzed by using ANOVA followed by the Bonferroni *t* test. A difference was considered statistically significant when p < 0.05.

## Results

### Evaluation of the therapeutic effect of PZP PDT: dependence on drug dose & irradiation dose

We first evaluated the therapeutic efficacy of PDT with PZP and xenon light in sarcoma S180 tumors in mice. PZP alone, at 5 and 20 mg/kg (free ZnPP equivalent), had no apparent antitumor effect (Figures 1 & 2), similar to our previous study [16]. However, when the tumors were irradiated with 400- to 700-nm xenon light at 36 J/cm^2^ (120 mW/cm^2^ for 5 min) at 24 and 48 h after the intravenous injection of PZP (5 mg/kg, ZnPP equivalent), a significant delay in tumor growth was observed: no apparent tumor growth occurred until day 23 after treatment (Figure 1A). Moreover, when we increased the dose of PZP to 20 mg/kg (ZnPP equivalent), a more marked dose-dependent PDT effect was achieved (Figure 1A). At both 5 and 10 mg/kg of ZnPP equivalent, one of five mice was tumor free at day 58 after treatment, respectively. With 20 mg/kg PZP (ZnPP equivalent), tumors turned pinkish to reddish and then blackish necrotic tissue appeared at 24 h after PDT; no sign of tumor growth was observed on day 21 after treatment (Figure 1Bi & Bii). Four of five mice were completely cured at the end of the experiment (Figure 1A). Moreover, in one mouse bearing two tumors, the tumor receiving light irradiation clearly demonstrated shrinkage, whereas the tumor that did not receive light treatment manifested no apparent effect (Figure 1Biii).

Also, no loss of body weight was observed in PDT treatment groups with PZP of 5 mg/kg and 10 mg/kg (ZnPP equivalent); body weight changes were similar to the growth of normal mice without tumor (Figure 1C). However, a trend of losing body weight gain was seen in the group of 20 mg/kg PZP (ZnPP equivalent), though no significant difference was found (Figure 1C). The difference of body weight between control group and treatment group is mostly attributed to the weight of tumors. Moreover, light irradiation at 36 J/cm^2^ alone also had a significant tumor-suppressive effect (Figure 1A). We thus reduced the intensity of light in the following studies and investigated the irradiation dose dependence in PDT.

With 20 mg/kg PZP (ZnPP equivalent), we varied the light intensity from 9 to 27 J/cm^2^ (30–90 mW/cm^2^ for 5 min for each treatment), and we clearly found an irradiation intensity-dependent effect (Figure 2A). Light irradiation at 27 J/cm^2^ alone had no effect on tumor growth. However, one PZP injection followed by two light irradiation (27 J/cm^2^) treatments at 24 and 48 h after PZP administration produced a marked antitumor effect (Figure 2A). Four of five mice were free of tumor at day 68 after PDT. In addition, the highest dose of this study (i.e., 20 mg/kg of PZP with 27 J/cm^2^ of irradiation) did not induce body weight loss of mice as compared with the growth of normal mice (inset of Figure 2A). These findings together with those shown in Figure 1 led us to conclude that 20 mg/kg PZP (ZnPP equivalent) and 27 J/cm^2^ light irradiation are the possible optimal dosages in this PDT.

We next investigated the effect of irradiation time versus light intensity on PDT. As seen in Figure 2B, with 20 mg/kg PZP (ZnPP equivalent) given intravenously, when the total irradiation dosage was fixed at 27 J/cm^2^ by modulating the irradiation intensity (30, 60 and 90 mW/cm^2^) and time period of irradiation (15, 7.5 and 5 min), we found that the higher light intensity (i.e., 90 mW/cm^2^) for a shorter exposure time (i.e., 5 min) achieved a better therapeutic effect: all mice were cured and evidenced no sign of tumor until at least 62 days after treatment, whereas growth or recurrence of tumors was observed when a lower intensity of light was applied for a longer time; significant difference (p < 0.05) was observed between 90 mW/cm^2^ group and 30 mW/cm^2^ group from 40 days after treatment (Figure 2B).

## Evaluation of the effect of PDT with PZP in carcinogen-induced breast tumors in rats

We also investigated the therapeutic effect of PZP in carcinogen-induced breast cancer in rats under the same conditions as those described above (one injection of PZP at 20 mg/kg of ZnPP equivalent and two light irradiation treatments at 27 J/cm^2^ for 5 min each). A dramatic therapeutic effect resulted (Figure 3A & B). Compared with untreated control rats, in which tumors grew quickly and achieved a diameter of 25–30 mm from a palpable tumor (˜10 mm in diameter) within 40 days (Figure 3B), rats receiving this PDT demonstrated a significant reduction in tumor size (Figure 3A & B). At days 50–60 after treatment, 80% of tumors had completely disappeared (Figure 3B).

Also, in a separate experiment, blue fluorescent light (Panasonic FL20S-B) with an emission wavelength matching the maximal absorption band (Soret band of ˜420 nm) of ZnPP was used to irradiate DMBA-induced tumors in a similar setting. Animals received 20 mg/kg PZP of ZnPP equivalent with 20 J/cm^2^ irradiation (2.8 mW/cm^2^ for 60 min) at 24, 48 and 72 h after PZP administration. A similar therapeutic effect was observed, as Figure 3C shows.

## *In vivo* fluorescence imaging of carcinogen-induced tumors after intravenous PZP injection

We previously found a potential use of PZP for *in vivo* imaging of tumors in an S180 solid tumor model [16]. In the present study, we continued investigating this possibility in DMAB-induced breast cancer in rats and in azoxymethane/DSS-induced colorectal cancer in mice. Tumors in both models demonstrated intense fluorescence, whereas normal tissues showed little fluorescence, at 24 h after intravenous injection of PZP (Figure 4). These findings demonstrated the potential applicability of PZP as a theranostic candidate with proper light irradiation.

## Generation of ^1^O_2_ from PZP after light irradiation: intact micelles versus disrupted micelles (i.e., with cell membrane components)

In our previous study, we showed that PZP formed a micellar complex in aqueous solutions in which no or very weak fluorescence was emitted because of the π-π interaction of chromophores and quenching, which resulted in no or slight generation of ^1^O_2_ and thus very little cytotoxicity [16]. However, disruption of micelles, for example, by detergents or organic solvents, led to generation of fluorescence and ^1^O_2_ after light irradiation and thus to cytotoxicity [16]. Because lipids are a major component of cell membranes, we hypothesized that PZP micelles may undergo disruption during transmembrane internalization as the micelles interact with the lipid bilayer. To support this hypothesis, we used the major cell membrane component lecithin and ESR spectroscopy to study the generation of ^1^O_2_ from PZP exposed to light. As Figure 5A illustrates, without lecithin, very little ^1^O_2_ signal could be detected even after 10 min of light irradiation, whereas in the presence of 100 μg/ml lecithin, ^1^O_2_ generation increased significantly in an irradiation time-dependent manner (Figure 5).

## Discussion

Targeted or tumor-selective accumulation is an essential consideration in cancer chemotherapy, because without such selectivity severe adverse side effects will occur. Dosage limits preclude additional increases in drug doses, which will result in an insufficient antitumor effect. So-called molecular target therapy, which usually focuses on specific molecules that are overexpressed in cancer cells, has recently received great attention. Many molecular target drugs have been developed and are used in clinical settings, but clinical results for those drugs are not encouraging, because only a 1- to 2-month extension of the usual 3- to 5-year overall survival has been achieved [24–27]. Problems associated with molecular target drugs probably relate to the intrinsic genetic diversity and extensive mutations in human solid tumors [28,29].

Macromolecular therapy or polymer therapeutics, however, should receive more attention because it applies to almost all solid tumors. Polymeric or nanoparticle drugs target tumor tissue selectively by means of the EPR effect, whose mechanism is based on the pathophysiological features of tumor tissue. Because of the EPR effect, macromolecular drugs including micelles, liposomes, protein-polymer conjugates and antibodies have superior tumor selectivity and *in vivo* pharmacokinetics compared with conventional small molecular anticancer drugs that are distributed indiscriminately to all tissues. Another important feature is the long retention time (days) of such nanosized drugs selectively in tumors, so that they exert improved antitumor effects with no or few adverse reactions [5,8–9,30–32].

The basic principle of PDT is the generation of cytotoxic ROS from photosensitizers after light irradiation. However, a prerequisite for PDT is achieving tumor-selective accumulation of photosensitizers so as to obtain satisfactory PDT effects. Otherwise, indiscriminate distribution of currently used low-molecular-weight photosensitizers will trigger severe side effects like many other small molecular anticancer drugs. Indeed, cancer patients receiving conventional PDT must stay in the dark during the treatment period, for more than a week or two, to avoid adverse effects. To overcome these drawbacks, we designed and synthesized a micellar form of the photosensitizer PZP, because such a fluorescent nanoprobe demonstrated tumor-selective accumulation. In contrast, free ZnPP showed no tumor-selective accumulation in a transplanted tumor model in mice [16]. This tumor- targeting potential of PZP was confirmed in a carcinogen-induced tumor model, which is far superior to the xenograft tumor model; we also achieved clear visualization of tumor-selective accumulation of the photosensitizer by using our *in vivo* imaging system (Figure 4). Thus, using PDT with PZP produced a marked antitumor effect. Not only transplanted tumors, but also carcinogen-induced breast cancer in rats manifested this impressive therapeutic effect (Figures 1–3).

Another key factor in PDT is the light source. Previously PDT used lasers (He/Ne) with an emission wavelength of >600 nm by taking advantage of the good tissue penetration and less chromophore absorption [33]. However, it always need special laser apparatus with high cost (˜$500,000 US), also the wavelength of the light sometimes does not match the maximal absorbance (Soret band) of photosensitizers (e.g., ˜410 nm for Photofrin). Therefore, generation of ^1^O_2_ may not be ideal or efficient enough to achieve the beneficial effect of PDT. Also, it has been expected that 410 nm light may give better results than 630 nm light for porphyrins [34]. Actually recently light emitting diodes have been widely used in PDT with more options of wavelength and lower cost compared with laser [33]. Along this line, in this study, we challenged a common xenon light source (Asahi Spectra) having the light spectrum of 400–700 nm, or a blue fluorescent tube (Panasonic FL20S-B) that emits light of about 420 nm, which matches the maximal absorbance of ZnPP (i.e., 420 nm) (see Supplementary Figure 1). We found that both the xenon light and the blue fluorescent light produced a sufficient PDT effect (Figures 1–3). Use of these light sources will be other options of PDT which may be beneficial in terms of the feasibility of PDT and the reduction in the cost of therapy. In addition, use of nonlaser light with bandpass filters is also a promising way to improve the specificity of light source, which will be further investigated in our future studies.

Compared to commonly used red light in PDT, one of the disadvantages of broad band xenon light and blue light is less tissue penetration. However, in the present study, we demonstrated a marked therapeutic effect, even against carcinogen-induced tumors, by using a fluorescent light with a peak wavelength of 420 nm (Figure 3C). These data suggest that the tumor-selective accumulation of photosensitizer (PZP) may partly compensate for the less penetration of blue light, namely the amount of light that penetrated these superficial tumors was sufficient to activate the and produce the PDT effect, only if the photosensitizer accumulated effectively and selectively in the tumor. And due to the shallow penetration this PDT using blue light or white light may be preferentially applied to the superficial and small tumors.

Tissue heating always accompanies with light irradiation, and hyperthermia may contribute partly to the PDT effect especially when the surface irradiance exceeds 200 mW/cm^2^. This is also a concern of using broad band xenon light. In this study, we filtered the light of >700 nm to minimize heat. In fact, we did find some tumor-suppressive effect of irradiation alone at higher dose (i.e., 36 J/cm^2^) (Figure 1A). However, when we lowered the irradiation dose to 27 J/cm^2^ or less, no apparent antitumor effect was observed (Figure 2), suggesting the results observed in this study is mostly the consequence of PDT, the effect of light exposure or tissue heating is marginal.

We should note that the light used in this study was relatively weak. In clinical PDT protocols with Photofrin, irradiation is usually performed at doses of 50–500 J/cm^2^, and many experimental studies of PDT commonly used a dose of approximately 100 J/cm^2^ [33–38]. In our study here, however, an irradiation dose of 27 J/cm^2^ almost completely cured the tumors, both transplanted and carcinogen-induced tumors (Figures 1–3). These findings thus support the beliefs that targeted accumulation of photosensitizers in tumors is the most important innovation in the present PDT and that a nonlaser light source, which covers or fits to the absorption (Soret) band of the photosensitizers, may be favorable for this PDT using PZP.

With regard to the timing of irradiation, in most experiments we irradiated tumors at 24 and 48 h after the intravenous injection of PZP, which would ensure the accumulation of the polymeric nanoprobe in tumors by virtue of the EPR mechanism [16]. When we examined the potential effect of irradiation intensity vs. irradiation time, with the same energy input, short high-intensity irradiation was better than long low-intensity irradiation (Figure 2B). Our explanation is that a burst of a large amount of ^1^O_2_ from higher power light may kill tumor cells more completely, whereas lower power light induces continuous but insufficient concentration of ^1^O_2_ which is associated with the recurrence of tumors at later stage of observation (Figure 2B). However, this findings are controversial to previous literatures, which showed lower irradiations with extended exposure time led to better antitumor activity of PDT primarily in relation to the tissue oxygen supply, namely high-power PDT may result in deficits in oxygen if the ^1^O_2_ generation rate exceeds the resupply of oxygen [39–42]. This inconsistency may be due to the much lower ^1^O_2_ quantum yields of polymeric ZnPP (i.e., 17%) [14] compared with conventional photosensitizers (i.e., lyserphyrin, 50–80%), so the oxygen consumption would not outpace its supply in the treatment protocol of this study. Under these circumstances, high-power light will exhibit more advantages than low-power light.

With respect to the *in vivo* mechanisms of ^1^O_2_ generation from PZP, PZP in the micellar form reportedly generated very little ^1^O_2_ after exposure to light; instead, the disruption of micelles was indispensable for efficient PDT [16]. How PZP micelles are disrupted *in vivo* in tumor tissues remains to be clarified. We envision a number of possible mechanisms, for example, the micelles may be disrupted by the amphipathic component of the cell membrane lipid bilayer, such as lecithin, during transmembrane internalization. As expected, we observed a time- and dose-dependent increase in ^1^O_2_ generation from PZP after lecithin treatment in our previous study [16] as well as in our present study (Figure 5). Moreover, once micelles are internalized by cells, in lysosomal conditions the micellar structure will be disrupted during phago-lysosomal fusion. In addition, many proteases such as cathepsin B are highly expressed in tumor tissues, which may help cleave the chemical bond between the prosthetic group and the polymers to release free drug (e.g., ZnPP) [43,44]. This process may also be involved in the *in vivo* mechanisms of PZP-induced PDT.

Furthermore, many recent studies focused on unique tumor microenvironments, beyond the EPR effect, to facilitate tumor distribution and intracellular uptake of macromolecular drugs. One typical strategy is to utilize the acidic and hypoxic conditions of tumors, for example, designing pH-sensitive bonds that would be cleaved and thereby release free drugs, predominantly in tumor tissues, thus achieving an improved antitumor effect [45]. Other strategies include the use of cleavable polypeptide linkers between polymers and drugs, for example, a cathepsin B-cleavable peptide linkage (glycylphenylalanylleucylglycine, GFLG) [46]. These issues will be addressed in further investigations of PZP.

## Conclusion & future perspective

We demonstrated here, by means of fluorescent tumor images *in vivo*, the tumor-selective accumulation of PZP and a therapeutic effect of PDT, in which a commonly used xenon light was applied, in different tumor models including carcinogen-induced tumors. A marked antitumor effect was achieved with only one injection of PZP followed by two or three light irradiation treatments. An irradiation dose of 27 J/cm^2^ was adequate to obtain sufficient regression of tumor. Laser (He/Ne) light was not required for this therapeutic protocol. For this PDT effect, three sequential processes may be critical: EPR-based tumor targeting, intracellular uptake of micelles followed by their disruption and generation of ^1^O_2_ by photoactivation of the photosensitizer ZnPP.

These findings suggest the potential utility of PZP as a theranostic PDT agent, with the use of a light source such as the xenon light of an endoscope, which could directly affect superficial tumors such as cancers of the esophagus, breast, lung, colon, rectum, urinary bladder and cervix. Future studies will focus on pathological analysis of tumors after PZP mediated PDT; evaluation of the pharmacokinetics and tumor-selective accumulation of PZP in different solid tumor models including carcinogen-induced tumors; investigations of the behaves and fate of PZP after accumulating in tumors, for example, the release/cleavage of free ZnPP from PZP conjugate; quantitative analysis of fluorescence images in tumors versus normal tissues, to further understand the mechanisms and efficacy of PZP medicated anticancer therapy and imaging.

**Executive summary**

**Background**

Wide application of photodynamic therapy (PDT) in cancer treatment has been limited, mostly attributing to the poor tumor-selective accumulation of commonly used small molecular weight photosensitizers.
Biocompatible macromolecular photosensitizer containing zinc protoporphyrin (ZnPP) predominantly accumulates in cancer after intravenous injection.

**Results**

In this study, the authors demonstrate the potential of *N*-(2-hydroxypropyl)­methacrylamide copolymer-conjugated ZnPP (PZP) in PDT and tumor imaging in different tumor models including carcinogen-induced tumors.
In the murine sarcoma S180 model with a xenon light of 400–700 nm, one PZP 20 mg/kg (ZnPP equivalent) dose with two or three treatments of light at an intensity of ≥27 J/cm^2^ caused necrosis and disappearance of most tumors (>70%).
Similar results were observed in 7,12-dimethylbenz[*a*]anthracene-induced breast cancer, with either the xenon light source or the commonly used blue fluorescent light.
PZP-based tumor imaging was also confirmed in the 7,12-dimethylbenz[*a*]anthracene-induced breast tumor and azoxymethane/dextran sulfate sodium-induced colon cancer models.

**Discussions**

These findings strongly support the potential application of PZP as a future nanomedicine for photodynamic cancer therapy and imaging.
Potential drawbacks of broad band xenon light and blue fluorescence light include higher tissue absorption and less penetration than red light; however, it may be preferential for ZnPP especially for superficial and small tumors.
Clinical translation of PZP-based PDT and imaging awaits optimization/modification of irradiation protocol and light source.
