# Supplementary material for: Photodynamic therapy and imaging based on tumor-targeted nanoprobe, polymer-conjugated zinc protoporphyrin
Source: Future Sci OA. 2015 Nov 1;1(3):FSO4. doi: 10.4155/fso.15.2 (PMC5137960; doi:10.4155/fso.15.2)
Supplement: Supplementary file 1 [file fso-01-4-s1.docx]

Supplemental data Figure S1

0

5

10

15

250

300

350

400

450

500

550

600

650

700

750

800

**Intensity (µW/cm^2^/nm)**

**Wavelength (nm)**

**Fig. S1.** Emission spectra of blue fluorescent tube (Panasonic FL20S-B)
